# Supplementary material for: KAT8-mediated H4K16ac is essential for sustaining trophoblast self-renewal and proliferation via regulating CDX2
Source: Nat Commun. 2024 Jul 3;15:5602. doi: 10.1038/s41467-024-49930-6 (PMC11222414; doi:10.1038/s41467-024-49930-6)
Supplement: Supplementary file 1 — Supplementary Information [file 41467_2024_49930_MOESM1_ESM.pdf]

KAT8-mediated H4K16ac is Essential for Sustaining Trophoblast Self-renewal and Proliferation via Regulating CDX2

Bi et al.

Description of Supplementary Information

Supplementary Figure 1-7.

Supplementary Table S1: The clinical characteristics of the pregnant women enrolled in this study

Supplementary Table S2: Primer list of constructing overexpression vector

Supplementary Table S3. Primer list of quantitative RT-PCR

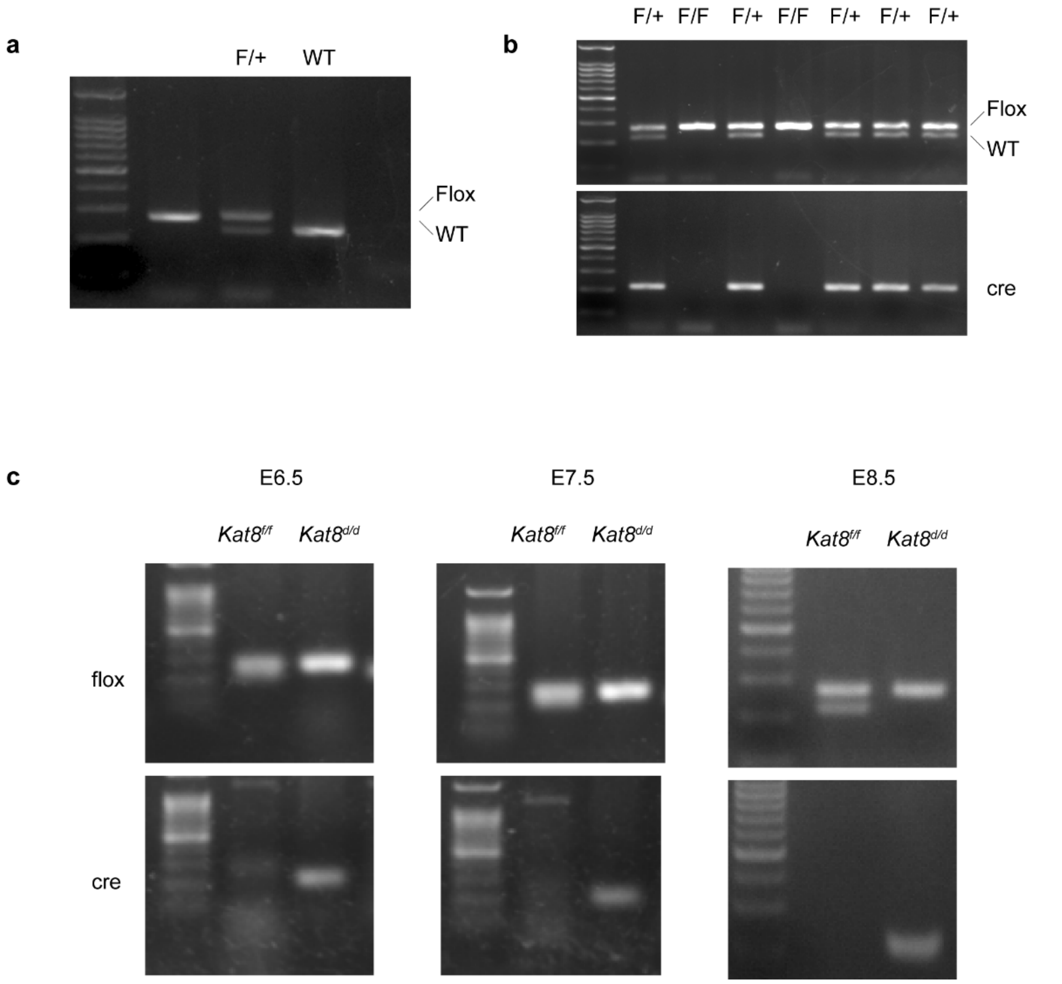

Supplementary Figure 1. Genotype analysis of the generated mouse model. (**a**) Genotype analysis of *Kat8*$^{f/+}$, *Kat8*$^{f/f}$, WT mouse. (**b**) Genotype analysis of the offspring from female *Kat8*$^{f/f}$ mice crossed with male *Kat8*$^{f/+}$;*Elf5-Cre* mice. (**c**) Genotype analysis of embryos obtained from the implantation site dissection of female *Kat8*$^{f/f}$ mice crossed with male *Kat8*$^{f/+}$;*Elf5-Cre* mice.

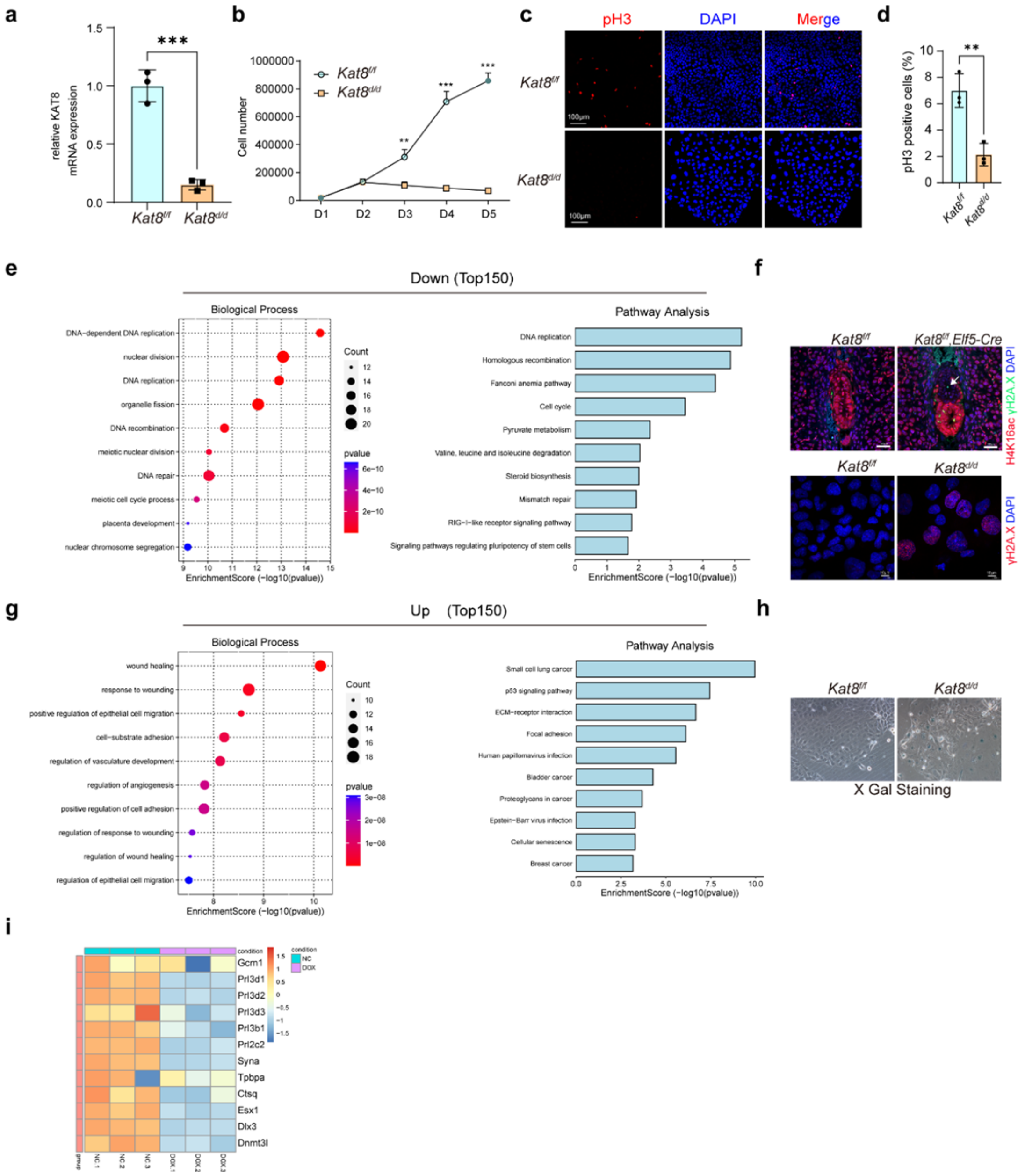

Supplementary Figure 2. Impaired trophoblast proliferation upon *Kat8* deletion. (**a**) Quantitative real-time PCR analysis of *Kat8* mRNA levels in *Kat8*$^{f/f}$ and *Kat8*$^{d/d}$ mTSCs. Data are representative of three independent experiments (n=3) and the values are normalized to ACTB. Two-tailed unpaired Student's t test. Error bars, mean ± SEM. P < 0.0001. Source data are provided as a Source Data file. (**b**) The analysis of cell proliferation between *Kat8*$^{f/f}$ and *Kat8*$^{d/d}$ mTSCs. Data are representative of three independent experiments (n=3). Two-tailed unpaired Student's t test. Error bars, mean ± SEM. P = 0.0042 (for D3), P = 0.0001 (for D4), P < 0.0001 (for D5). Source data are

provided as a Source Data file. (**c**) IF staining images of pH3 in *Kat8*$^{f/f}$ and *Kat8*$^{d/d}$ mTSCs. (**d**) The quantitative results of C. Data are representative of three independent experiments (n=3). Two-tailed unpaired Student's t test. Error bars, mean ± SEM. P = 0.0053. (**e**) Gene Ontology analysis for top 150 downregulated genes. (**f**) IF staining images of γH2AX and H4K16ac in *Kat8*$^{f/f}$, *Kat8*$^{d/d}$ embryos and mTSCs. (**g**) Gene Ontology analysis for top 150 upregulated genes. (**h**) X gal staining in *Kat8*$^{f/f}$ and *Kat8*$^{d/d}$ mTSCs. (**i**) Heatmap of differentiation of mTSCs associated genes differentially regulated between *Kat8*$^{f/f}$ and *Kat8*$^{d/d}$ mTSCs.

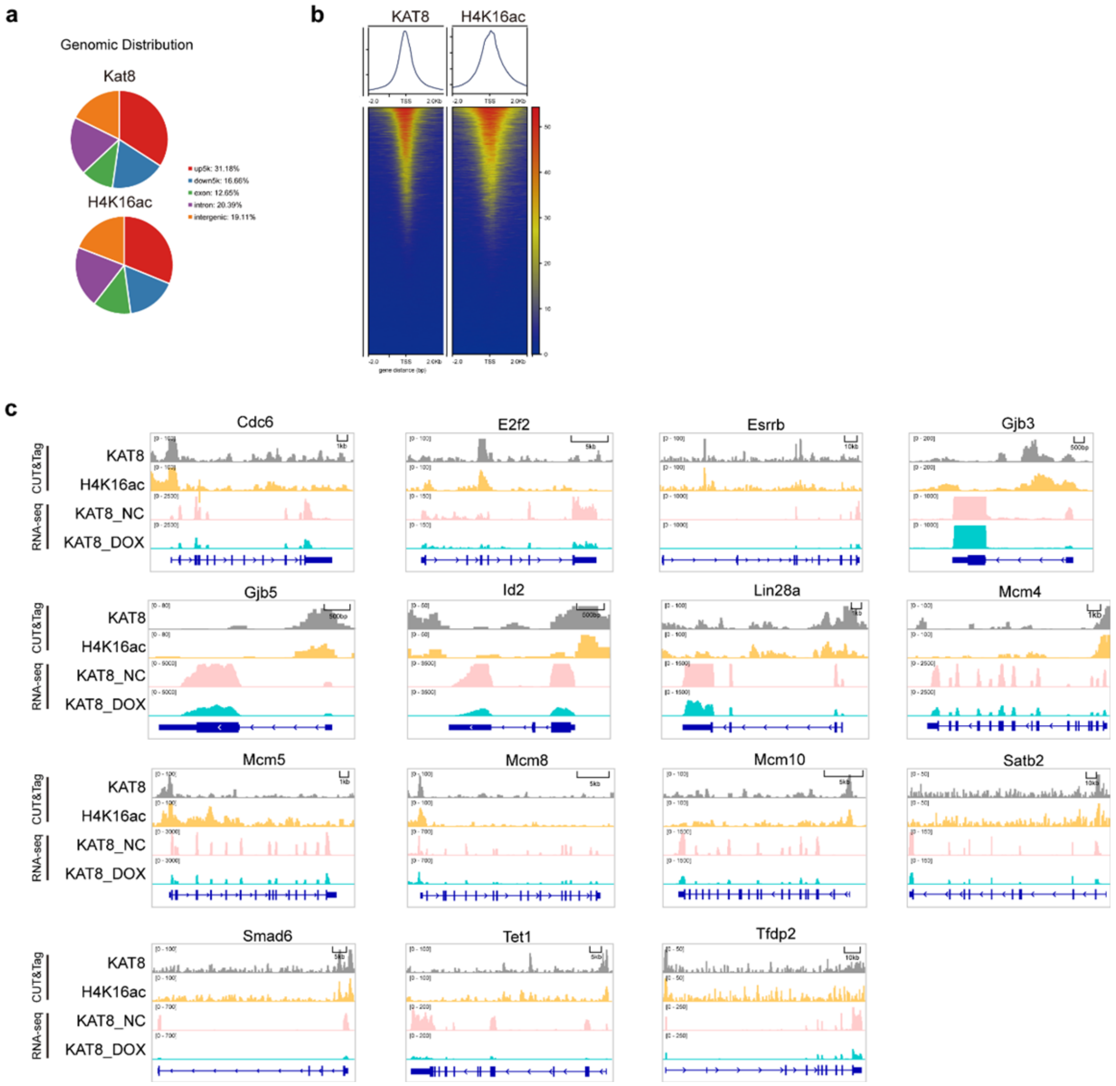

Supplementary Figure 3. Impaired trophoblast proliferation upon *Kat8* deletion. (**a**) Genomic distribution of KAT8 and H4K16ac peaks in mTSCs. (**b**) Coverage profiles for KAT8 and H4K16ac. (**c**) Genome browser view of CUT&Tag-seq signals and RNA-seq tracks for KAT8 and H4K16ac target genes in mTSCs

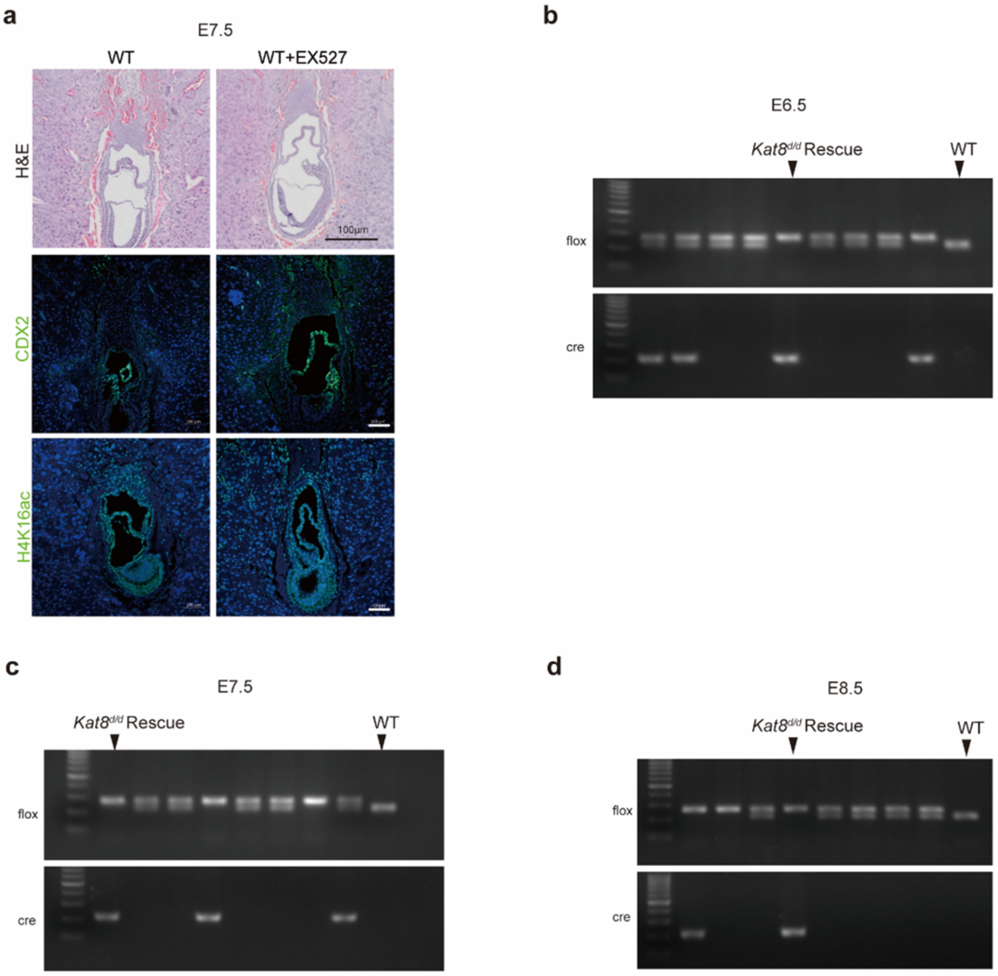

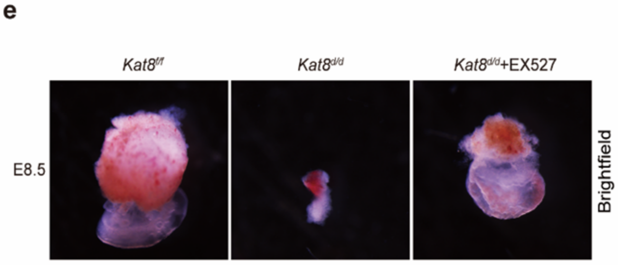

f

No. Rescue embryo

Stage	No. *Kat8*$^{d/d}$ embryo	No. Rescue embryo
E6.5	8	4
E7.5	8	3
E8.5	6	2

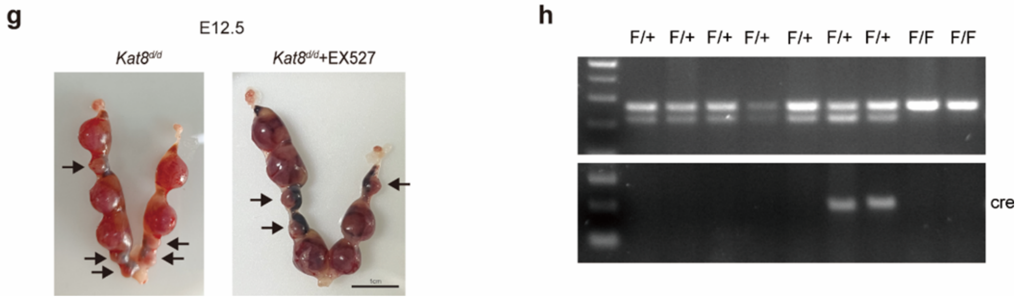

Supplementary Figure 4. Partial restoration of abnormal placental development caused by KAT8 knockout through EX527 intervention. (**a**) H&E staining, immunofluorescent staining of CDX2 and H4K16ac in WT mice treated with 20mg/kg EX527 at E7.5. (**b**) Genotype analysis of embryos from female *Kat8*$^{f/f}$ mice crossed with male *Kat8*$^{f/+}$;*Elf5-Cre* mice after treatment with EX527 at E6.5. (**c**) Genotype analysis of embryos from female *Kat8*$^{f/f}$ mice crossed with male *Kat8*$^{f/+}$;*Elf5-Cre* mice after treatment with EX527 at E7.5. (**d**) Genotype analysis of embryos from female *Kat8*$^{f/f}$ mice crossed with male *Kat8*$^{f/+}$;*Elf5-Cre* mice after treatment with EX527 at E8.5. (**e**) Brightfield images of embryos from female *Kat8*$^{f/f}$ mice crossed with male *Kat8*$^{f/+}$;*Elf5-Cre* mice after restoration treatment with EX527. (**f**) The number of KAT8 knockout embryos and the number of rescued embryos in female *Kat8*$^{f/f}$ mice crossed with male *Kat8*$^{f/+}$;*Elf5-Cre* mice after restoration treatment with EX527. Rescued embryos were defined as *Kat8*$^{d/d}$ embryos exhibiting CDX2+ trophoblast at E6.5 and E7.5, and AP2γ+ trophoblast at E8.5. (**g**) Implantation sites in female *Kat8*$^{f/f}$ mice crossed with male *Kat8*$^{f/+}$ *Elf5-Cre* mice at E12.5 with EX527 or without EX527. (**h**) Genotype analysis of offspring from female *Kat8*$^{f/f}$ mice crossed with male *Kat8*$^{f/+}$;*Elf5-Cre* mice after treatment with EX527.

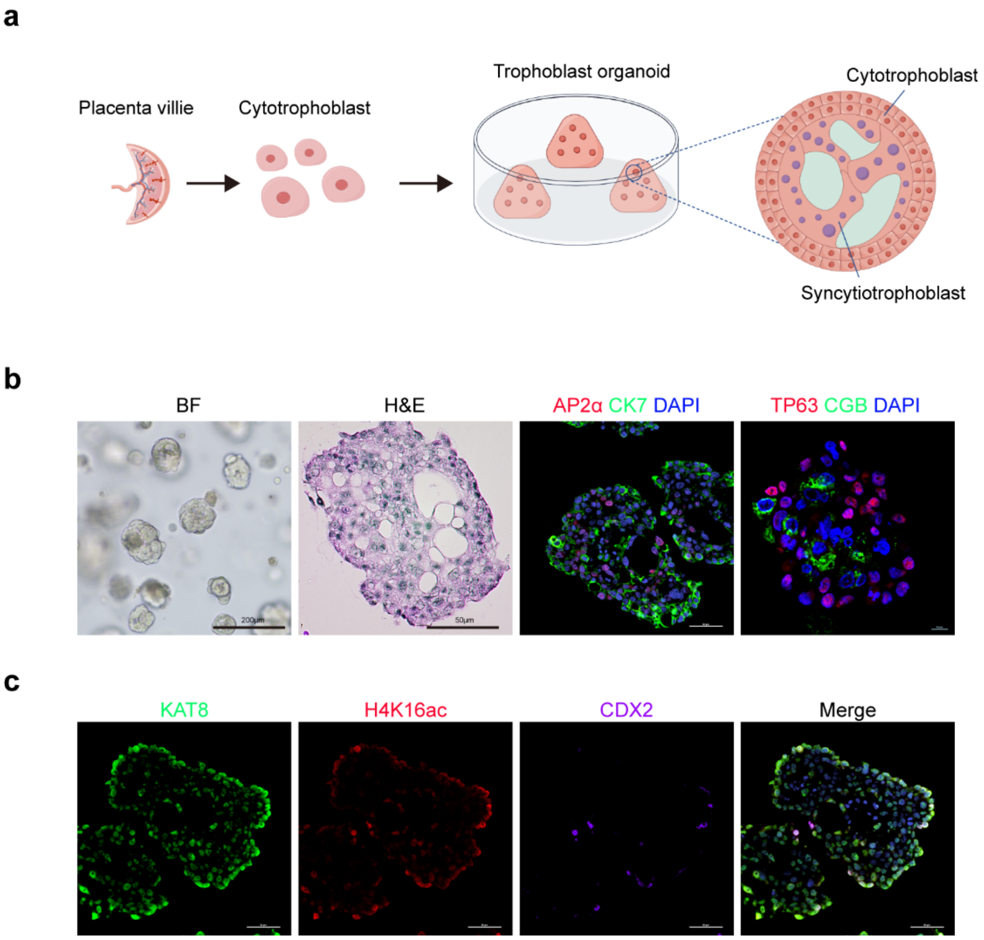

Supplementary Figure 5. Generating human trophoblast organoid. (**a**) Schematic representation illustrating the workflow for generating human placenta-derived organoids. The graphic elements were created by figdraw. (**b**) Brightfield images, H&E staining, and IF staining of AP2α, CK7, TP63, and CGB in hTOs. (**c**) IF staining of KAT8, H4K16ac and CDX2 in hTOs.

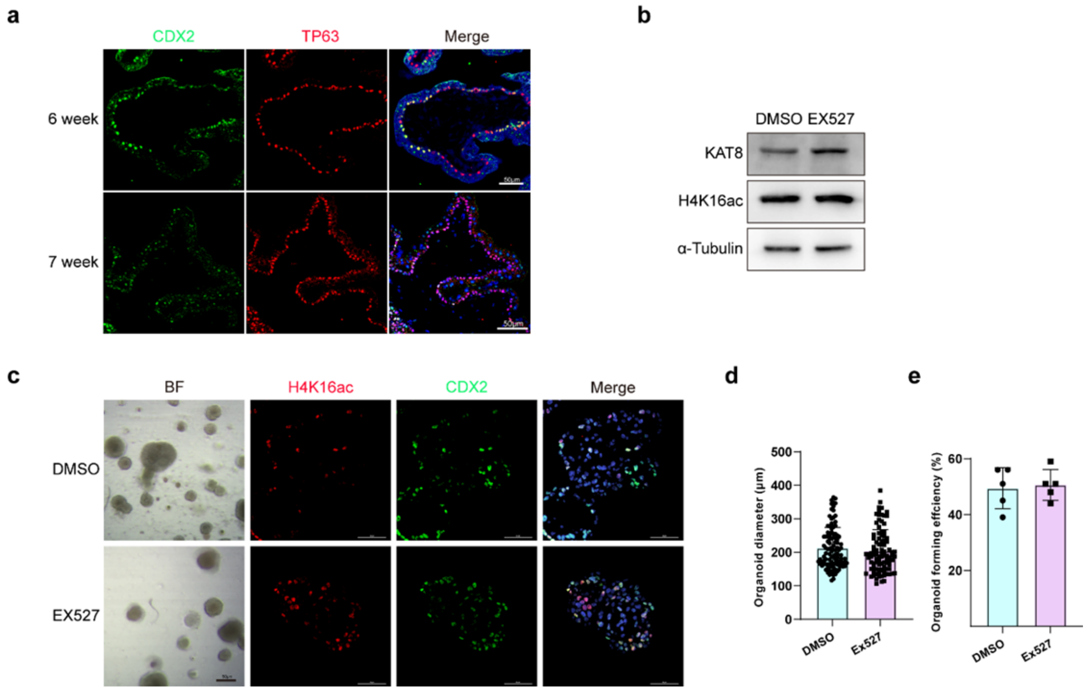

Supplementary Figure 6. Assessing the impact of EX527. (**a**) Immunofluorescent staining of CDX2 in villi from 6 and 7 weeks. (**b**) Immunoblot analysis of KAT8 and H4K16ac in hTOs treated with or without EX527. (**c**) Brightfield images, and immunofluorescent staining of KAT8 and H4K16ac in hTOs with or without EX527. (**d**) The quantification results of organoid diameter for hTOs in indicated groups. Each dot in the column represents one TO (n=105, DMSO; n=93, EX527). Source data are provided as a Source Data file. (**e**) The quantification results of organoid forming efficiency for hTOs in indicated groups. Data are representative of five independent experiments (n=5). Source data are provided as a Source Data file.

Fig. 2b

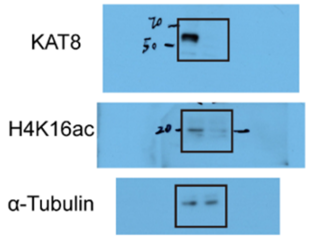

Fig. 3e

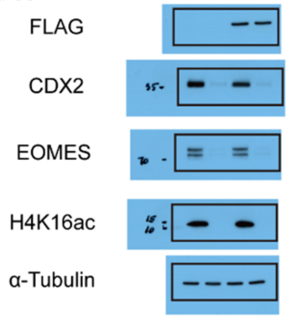

Fig. 4l

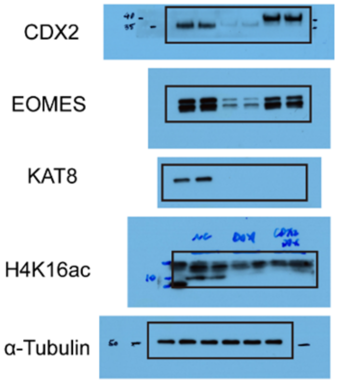

Fig. 3b

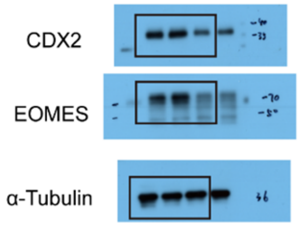

Fig. 4h

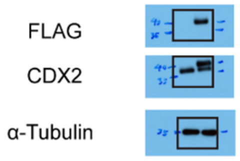

Fig. 5b

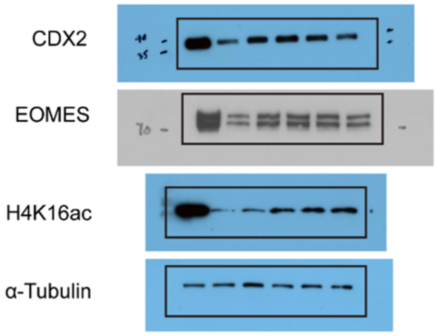

Fig. 6d

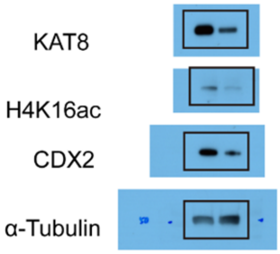

Fig. 6b

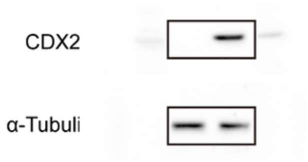

Fig. 6g

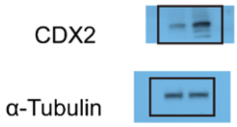

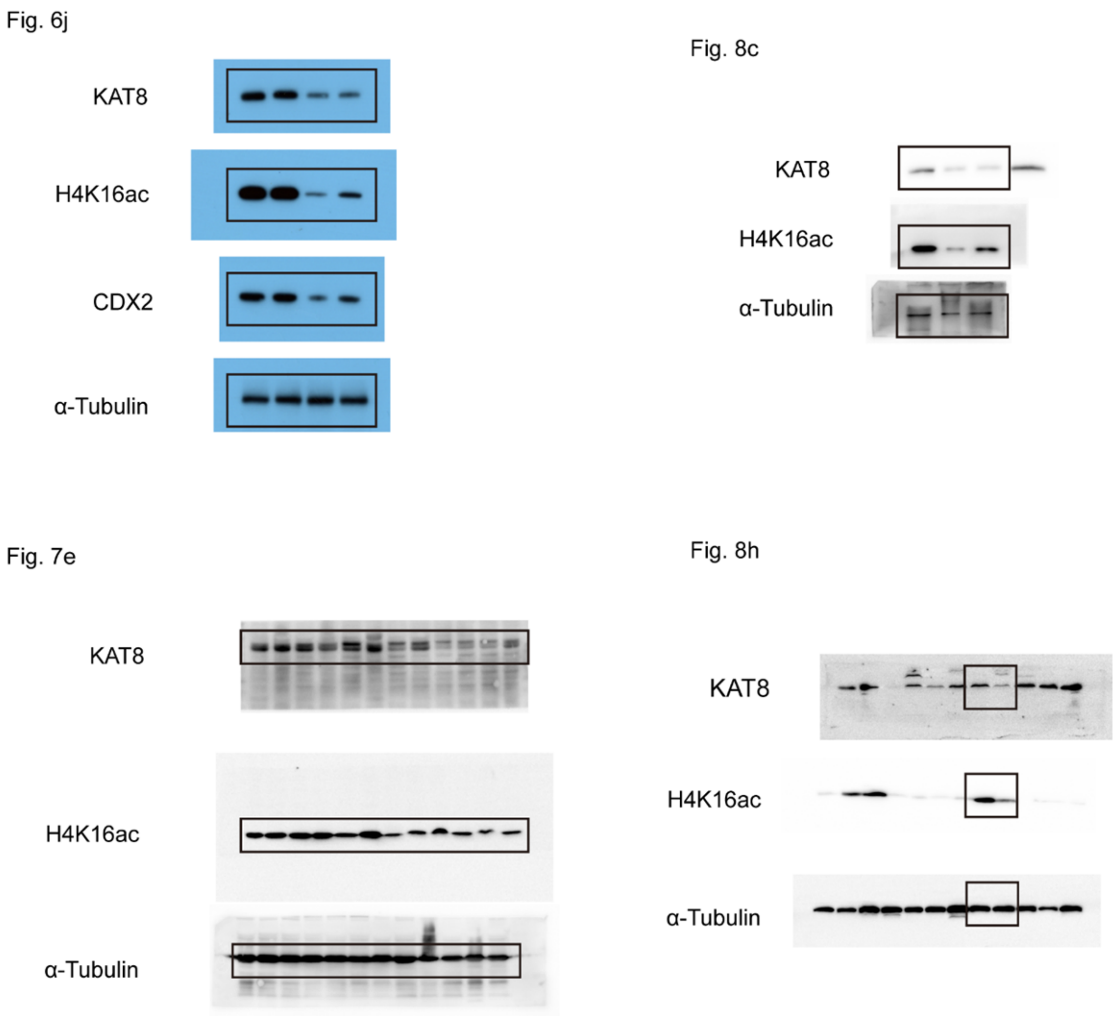

Supplementary Figure 7. Unprocessed images of immunoblotting.

PVDF membranes were cut into several small pieces to incubate with different antibodies for immunoblotting. Black boxes indicate images showed in relevant figures.

Supplementary Table S1. The clinical characteristics of the pregnant women enrolled in this study

	Con	RPL	P value
Total sample	28	24	
Maternal age(year)	31.2±0.7	33.0±1.1	ns
Gestational age(day)	53±1.3	53±1.9	ns
Number of spontaneous abortions	n/a	2.4±0.18	n/a

Two-tailed unpaired Student's t test. Data are mean ± SEM. D&C, dilatation and curettage; ns, not statistically significant;

Supplementary Table S2. Primer list of constructing overexpression vector

Gene	Forward Primer
mCdx2	CCAGTAACGTGGCGCGCCTTAtcactgggtgacagtg
hCDX2	ACTAGAGGATCTATTTCCGGTGAATTCgccaccatgtacgtgagctacctc
KAT8	ACTGCTTACTGGCTTATCGGTCGACatggcggcacagggagctacag
Gene	Reverse Primer
mCdx2	CGATGACAAGGCTAGCatgtacgtgagctaccttc
hCDX2	GCGGCTTCGGCCAGTAACGTGGATCCtcactgggtgacggtggggtttagc
KAT8	CCGTCATGGTCTTTGTAGTCGCTAGCAGAGGAGCCcttcttggaaagcttgactt

Supplementary Table S3. Primer list of quantitative RT-PCR

quantitative RT-PCR primer for mTSCs		
Gene	Forward Primer	Reverse Primer
Cdx2	TCCTGCTGACTGCTTTCTGA	CCCTTCCTGATTTGTGGAGA
Eomes	CCTGGTGGTGTTTTGTTGTG	TTTAATAGCACCGGGCACTC
Pl1	TGGAGCCTACATTGTGGTGG	TGGCAGTTGGTTTGGAGGA
Pl2	CCAACGTGTGATTGTGGTGT	TGCCACCATGTGTTTCAGAG
Actb	TGTTACCAACTGGGACGACA	GGGGTGTTGAAGGTCTCAAA
Kat8	ATGGGTGGACAAGAACCGAC	GTTTCGAGTGATCTTGCGCTC
Esrrb	GCAGTCCTTCGTGCTGTCTCA	TACATGGGACTGGATGGGAGAT
Elf5	ACAAGCTTGATGCCAACTGC	CCTTGCGAGCGAATGTTCTG
Ctsq	CAGGCAAACTGATCCCACTGA	CAACGACAGCCTCTATTGCCTT
Plf	CTCAGAGACAAAAGCCCCATGA	TGAGCCTGGCTTGTTCCTTG
Ly6a	AGGAGGCAGCAGTTATTGTGG	CGTTGACCTTAGTACCCAGGA
quantitative RT-PCR primer for hTOs		
Gene	Forward Primer	Reverse Primer
CDX2	GACGTGAGCATGTACCCTAGC	GCGTAGCCATTCCAGTCCT
KAT8	GTCACGGTGGAGATCGGAGA	CCCTCCTGGTCGTTCACTC

ACTB	CTACCTCATGAAGATCCTCACCGA	TTCTCCTTAATGTCACGCACGATT
