# Supplementary material for: KAT8-mediated H4K16ac is essential for sustaining trophoblast self-renewal and proliferation via regulating CDX2
Source: Nat Commun. 2024 Jul 3;15:5602. doi: 10.1038/s41467-024-49930-6 (PMC11222414; doi:10.1038/s41467-024-49930-6)
Supplement: Supplementary file 3 — Reporting Summary [file 41467_2024_49930_MOESM3_ESM.pdf]

nature portfolio

Corresponding author(s): Shuang Zhang

Last updated by author(s): 2024-4-19

Reporting Summary

Nature Portfolio wishes to improve the reproducibility of the work that we publish. This form provides structure for consistency and transparency in reporting. For further information on Nature Portfolio policies, see our Editorial Policies and the Editorial Policy Checklist.

Statistics

For all statistical analyses, confirm that the following items are present in the figure legend, table legend, main text, or Methods section.

n/a	Confirmed	
☐	☒	The exact sample size (*n*) for each experimental group/condition, given as a discrete number and unit of measurement
☐	☒	A statement on whether measurements were taken from distinct samples or whether the same sample was measured repeatedly
☐	☒	The statistical test(s) used AND whether they are one- or two-sided<br>*Only common tests should be described solely by name; describe more complex techniques in the Methods section.*
☒	☐	A description of all covariates tested
☒	☐	A description of any assumptions or corrections, such as tests of normality and adjustment for multiple comparisons
☐	☒	A full description of the statistical parameters including central tendency (e.g. means) or other basic estimates (e.g. regression coefficient) AND variation (e.g. standard deviation) or associated estimates of uncertainty (e.g. confidence intervals)
☐	☒	For null hypothesis testing, the test statistic (e.g. *F*, *t*, *r*) with confidence intervals, effect sizes, degrees of freedom and *P* value noted<br>*Give P values as exact values whenever suitable.*
☒	☐	For Bayesian analysis, information on the choice of priors and Markov chain Monte Carlo settings
☒	☐	For hierarchical and complex designs, identification of the appropriate level for tests and full reporting of outcomes
☒	☐	Estimates of effect sizes (e.g. Cohen's *d*, Pearson's *r*), indicating how they were calculated

Our web collection on statistics for biologists contains articles on many of the points above.

Software and code

Policy information about availability of computer code

Data collection	OLYMUPUS VS-ASW 2.9 (Olympas), Zen 2.3 (blue edition)(Zeiss), NIS-Elements AR 5.21.00 (Nikon), Bio-Rad CFX Manager 3.1(BioRad) were commercially available software used in data collection.
Data analysis	Microsoft Excel (professional plus 2016)<br>Fiji image J2 software (version1.8.0)<br>GraphPad Prism 6 (GraphPad Software)<br>Trim Galore (version 0.6.4)<br>STAR (version 2.7.3a)<br>featureCounts (version 2.0.0)<br>DEseq2 (version 1.30.1)<br>Bowtie2 (version 2.3.5.1)<br>Xtail package (version 1.1.5)<br>MACS2 (version 2.1.2)<br>BEDTools' intersect (version 2.28.0)<br>igvtools (version 2.9.4)<br>HOMER (version 4.11.1)<br>BEDTools' shuffleBed (version 2.28.0)<br>Metascape (http://metascape.org)<br>GSEA software (version 4.1.0)

Heatmap plots were plotted on http://www.bioinformatics.com.cn

For manuscripts utilizing custom algorithms or software that are central to the research but not yet described in published literature, software must be made available to editors and reviewers. We strongly encourage code deposition in a community repository (e.g. GitHub). See the Nature Portfolio guidelines for submitting code & software for further information.

Data

Policy information about availability of data

All manuscripts must include a data availability statement. This statement should provide the following information, where applicable:

- Accession codes, unique identifiers, or web links for publicly available datasets
- A description of any restrictions on data availability
- For clinical datasets or third party data, please ensure that the statement adheres to our policy

Provide your data availability statement here.

Research involving human participants, their data, or biological material

Policy information about studies with human participants or human data. See also policy information about sex, gender (identity/presentation), and sexual orientation and race, ethnicity and racism.

Reporting on sex and gender	female
Reporting on race, ethnicity, or other socially relevant groupings	ethnic Han
Population characteristics	Healthy women who terminated pregnancy for other reasons; RPL patients.
Recruitment	Patients presenting with the following conditions were systematically excluded from the study cohort: (1) congenital anomalies affecting the reproductive system; (2) aberrant karyotypes observed in both parents and abortuses; (3) endocrine or metabolic dysfunctions; (4) autoimmune disorders; (5) coexisting major illnesses; (6) inadequate pharmaceutical intervention, as well as exposure to hazardous chemicals or radiation.
Ethics oversight	This study has been approved by the Ethics Committee of the Third Affiliated Hospital of Guangzhou Medical University, with approval number 2018002, and informed consent was obtained from all participants.

Note that full information on the approval of the study protocol must also be provided in the manuscript.

Field-specific reporting

Please select the one below that is the best fit for your research. If you are not sure, read the appropriate sections before making your selection.

☒ Life sciences ☐ Behavioural & social sciences ☐ Ecological, evolutionary & environmental sciences

For a reference copy of the document with all sections, see nature.com/documents/nr-reporting-summary-flat.pdf

Life sciences study design

All studies must disclose on these points even when the disclosure is negative.

Sample size	No statistical method was used to predetermine the sample size. Sample size was determined to reflect biological and technical variance of the investigated parameters based on previously published literature (10.1016/j.jgg.2022.03.005). For all histology, immunofluorescence, western blot, immunoprecipitation, and qPCR experiments, we performed at least three independent biological replicates.
Data exclusions	No data were excluded from analyses.
Replication	All experiments were performed for at least 3 independent biological replicates except for CUT&Tag with 2 independent biological replicates.
Randomization	Samples and cells were randomly allocated into groups. Mice with comparable age and body size were randomly selected and divided into experimental groups.
Blinding	Investigators were blinded to group allocation of experiment and data analysis.

Reporting for specific materials, systems and methods

We require information from authors about some types of materials, experimental systems and methods used in many studies. Here, indicate whether each material, system or method listed is relevant to your study. If you are not sure if a list item applies to your research, read the appropriate section before selecting a response.

Materials & experimental systems

n/a	Involved in the study
☐	☒ Antibodies
☐	☒ Eukaryotic cell lines
☐	☐ Palaeontology and archaeology
☐	☒ Animals and other organisms
☒	☐ Clinical data
☐	☐ Dual use research of concern
☐	☐ Plants

Methods

n/a	Involved in the study
☐	☒ ChIP-seq
☐	☐ Flow cytometry
☐	☐ MRI-based neuroimaging

Antibodies

Antibodies used

For WB, KAT8 (Abcam, ab200660), H4K16ac (Millipore, 07-329), CDX2 (Abcam, ab76541), EOMES (Abcam, ab23345), α-Tubulin (Proteintech, 11224-1-AP); For IF, KAT8 (Abcam, ab200660), H4K16ac (Millipore, 07-329), Ki67 (Invitrogen, 14-5698-82), γH2A.X (Millipore, 05-636), CDX2 (Abcam, ab76541), EOMES (Abcam, ab23345), AP2γ (Santa Cruz, SC12762), CK8 (DSHB, AB_531826), AP2α (Abcam, ab108311), CGB (Abcam, ab9582); For CUT&Tag, KAT8 (Abclonal, A3390), H4K16ac (Millipore, 07-329)H4K16ac (Millipore 07-329)

Validation

validation of the commercial antibodies were done by the manufacturers.
Below shows the relevant citations listed on the suppliers' websites for antibodies used in this article:
KAT8, (Abcam, ab200660), https://pubmed.ncbi.nlm.nih.gov/33657400/
H4K16ac (Millipore, 07-329), https://pubmed.ncbi.nlm.nih.gov/23990607/
CDX2 (Abcam, ab76541), https://pubmed.ncbi.nlm.nih.gov/35266581/
EOMES (Abcam, ab23345), https://pubmed.ncbi.nlm.nih.gov/35046122/
α-Tubulin (Proteintech, 11224-1-AP), https://pubmed.ncbi.nlm.nih.gov/36650286/
Ki67 (Invitrogen, 14-5698-82), https://pubmed.ncbi.nlm.nih.gov/26949251/
γH2A.X (Millipore, 05-636), https://pubmed.ncbi.nlm.nih.gov/26080406/
AP2γ (Santa Cruz, SC12762), https://pubmed.ncbi.nlm.nih.gov/37438660/
CK8 (DSHB, AB_531826), https://pubmed.ncbi.nlm.nih.gov/33817835/
CGB (Abcam, ab9582), https://pubmed.ncbi.nlm.nih.gov/36241723/
KAT8 (Abclonal, A3390), https://pubmed.ncbi.nlm.nih.gov/37240065/

Eukaryotic cell lines

Policy information about cell lines and Sex and Gender in Research

Cell line source(s)

H9 human embryonic stem cell (ESCs) lines used in this study (a gift from Professor Fan Yong and Dr. Chaohui Li, Guangzhou Medical University, China). Sex of H9-hESCs is female.

Authentication

H9-hESCs were authenticated by STR profiling.

Mycoplasma contamination

All cell lines were routinely tested negative for mycoplasma

Commonly misidentified lines
(See ICLAC register)

No misidentified cell lines were used in this study

Palaeontology and Archaeology

Specimen provenance

Provide provenance information for specimens and describe permits that were obtained for the work (including the name of the issuing authority, the date of issue, and any identifying information). Permits should encompass collection and, where applicable, export.

Specimen deposition

Indicate where the specimens have been deposited to permit free access by other researchers.

Dating methods

If new dates are provided, describe how they were obtained (e.g. collection, storage, sample pretreatment and measurement), where they were obtained (i.e. lab name), the calibration program and the protocol for quality assurance OR state that no new dates are provided.

☐ Tick this box to confirm that the raw and calibrated dates are available in the paper or in Supplementary Information.

Ethics oversight

Identify the organization(s) that approved or provided guidance on the study protocol, OR state that no ethical approval or guidance was required and explain why not.

Note that full information on the approval of the study protocol must also be provided in the manuscript.

Animals and other research organisms

Policy information about studies involving animals; ARRIVE guidelines recommended for reporting animal research, and Sex and Gender in Research

Laboratory animals	All mice in this study were C57BL6 strains. Mice were maintained under specific pathogen-free conditions of a 12h light/dark cycle at controlled temperature (20-25°C) and humidity (50-70%) and were provided with food and water ad libitum in the Animal Care Facility at National Institute of Biological Sciences, Beijing. Elf5-Cre mice were C57BL6 strains, which is a gift from Wang Haibin Laboratory and these adult mice were used to mate with Kat8 fl/fl mice to generate Kat8-cko embryo.
Wild animals	No wild animals were used in the study.
Reporting on sex	Elf5-Cre/Kat8 f+ were male, Kat8 f/f were female
Field-collected samples	No field-collected samples were used in the study.
Ethics oversight	All animal experiments were approved by the Chinese Ministry of Health national guidelines and performed following institutional regulations of Institutional Animal Care and Use Committee at the National Institute of Biological Sciences, Beijing.

Note that full information on the approval of the study protocol must also be provided in the manuscript.

Dual use research of concern

Policy information about dual use research of concern

Hazards

Could the accidental, deliberate or reckless misuse of agents or technologies generated in the work, or the application of information presented in the manuscript, pose a threat to:

No	Yes	
☐	☐	Public health
☐	☐	National security
☐	☐	Crops and/or livestock
☐	☐	Ecosystems
☐	☐	Any other significant area

Experiments of concern

Does the work involve any of these experiments of concern:

No	Yes	
☐	☐	Demonstrate how to render a vaccine ineffective
☐	☐	Confer resistance to therapeutically useful antibiotics or antiviral agents
☐	☐	Enhance the virulence of a pathogen or render a nonpathogen virulent
☐	☐	Increase transmissibility of a pathogen
☐	☐	Alter the host range of a pathogen
☐	☐	Enable evasion of diagnostic/detection modalities
☐	☐	Enable the weaponization of a biological agent or toxin
☐	☐	Any other potentially harmful combination of experiments and agents

Plants

Seed stocks

Report on the source of all seed stocks or other plant material used. If applicable, state the seed stock centre and catalogue number. If plant specimens were collected from the field, describe the collection location, date and sampling procedures.

Novel plant genotypes

Describe the methods by which all novel plant genotypes were produced. This includes those generated by transgenic approaches, gene editing, chemical/radiation-based mutagenesis and hybridization. For transgenic lines, describe the transformation method, the number of independent lines analyzed and the generation upon which experiments were performed. For gene-edited lines, describe the editor used, the endogenous sequence targeted for editing, the targeting guide RNA sequence (if applicable) and how the editor was applied.

Authentication

Describe any authentication procedures for each seed stock used or novel genotype generated. Describe any experiments used to assess the effect of a mutation and, where applicable, how potential secondary effects (e.g. second site T-DNA insertions, mosiacism, off-target gene editing) were examined.

ChIP-seq

Data deposition

☒ Confirm that both raw and final processed data have been deposited in a public database such as GEO.

☒ Confirm that you have deposited or provided access to graph files (e.g. BED files) for the called peaks.

Data access links<br>*May remain private before publication.*	https://ngdc.cncb.ac.cn/gsa.
Files in database submission	CRR1083159 mTSC_KAT8-Kat8_abc<br>CRR1083160 mTSC-KAT8-H4K16ac<br>CRR1083115 mTSC_KAT8-Kat8_abc<br>CRR1083116 mTSC-KAT8-H4K16ac
Genome browser session<br>(e.g. UCSC)	*Provide a link to an anonymized genome browser session for "Initial submission" and "Revised version" documents only, to enable peer review. Write "no longer applicable" for "Final submission" documents.*

Methodology

Replicates	For KAT8 CUT&Tag: Two biological replicates were generated.<br>For H4K16ac CUT&Tag: Two biological replicates were generated.
Sequencing depth	For KAT8 CUT&Tag: Sequencing depth: 70 million. Uniquely mapped reads: 80%. Read length: 50bp. Single-end. For KAT8 CUT&Tag: Sequencing depth: 60-70 million. Uniquely mapped reads: 80%. Read length: 42+42bp. Paired-end.
Antibodies	KAT8 (Abclonal, A3390), H4K16ac (Millipore, 07-329)
Peak calling parameters	macs2 callpeak -t ${id}.bam -n ${i} -f BAMPE -g mm
Data quality	High-confidence, reproducible peaks among replicates were identified using the irreproducible discovery rate (IDR) framework with a global IDR < 0.05.
Software	bowtie2: v2.3.4.3, https://bowtie-bio.sourceforge.net/bowtie2/index.shtml<br>macs2, v2.1.1.20160309, https://github.com/macs3-project/MACS/wiki/Install-macs2<br>IDR: v2.0.2, https://github.com/nboley/idr

Flow Cytometry

Plots

Confirm that:

☐ The axis labels state the marker and fluorochrome used (e.g. CD4-FITC).

☐ The axis scales are clearly visible. Include numbers along axes only for bottom left plot of group (a 'group' is an analysis of identical markers).

☐ All plots are contour plots with outliers or pseudocolor plots.

☐ A numerical value for number of cells or percentage (with statistics) is provided.

Methodology

Sample preparation	*Describe the sample preparation, detailing the biological source of the cells and any tissue processing steps used.*
Instrument	*Identify the instrument used for data collection, specifying make and model number.*
Software	*Describe the software used to collect and analyze the flow cytometry data. For custom code that has been deposited into a community repository, provide accession details.*
Cell population abundance	*Describe the abundance of the relevant cell populations within post-sort fractions, providing details on the purity of the samples and how it was determined.*
Gating strategy	*Describe the gating strategy used for all relevant experiments, specifying the preliminary FSC/SSC gates of the starting cell population, indicating where boundaries between "positive" and "negative" staining cell populations are defined.*

☐ Tick this box to confirm that a figure exemplifying the gating strategy is provided in the Supplementary Information.

Magnetic resonance imaging

Experimental design

Design type	*Indicate task or resting state; event-related or block design.*
Design specifications	*Specify the number of blocks, trials or experimental units per session and/or subject, and specify the length of each trial or block (if trials are blocked) and interval between trials.*
Behavioral performance measures	*State number and/or type of variables recorded (e.g. correct button press, response time) and what statistics were used to establish that the subjects were performing the task as expected (e.g. mean, range, and/or standard deviation across subjects).*

Acquisition

Imaging type(s)	*Specify: functional, structural, diffusion, perfusion.*
Field strength	*Specify in Tesla*
Sequence & imaging parameters	*Specify the pulse sequence type (gradient echo, spin echo, etc.), imaging type (EPI, spiral, etc.), field of view, matrix size, slice thickness, orientation and TE/TR/flip angle.*
Area of acquisition	*State whether a whole brain scan was used OR define the area of acquisition, describing how the region was determined.*

Diffusion MRI ☐ Used ☐ Not used

Preprocessing

Preprocessing software	*Provide detail on software version and revision number and on specific parameters (model/functions, brain extraction, segmentation, smoothing kernel size, etc.).*
Normalization	*If data were normalized/standardized, describe the approach(es): specify linear or non-linear and define image types used for transformation OR indicate that data were not normalized and explain rationale for lack of normalization.*
Normalization template	*Describe the template used for normalization/transformation, specifying subject space or group standardized space (e.g. original Talairach, MNI305, ICBM152) OR indicate that the data were not normalized.*
Noise and artifact removal	*Describe your procedure(s) for artifact and structured noise removal, specifying motion parameters, tissue signals and physiological signals (heart rate, respiration).*
Volume censoring	*Define your software and/or method and criteria for volume censoring, and state the extent of such censoring.*

Statistical modeling & inference

Model type and settings	*Specify type (mass univariate, multivariate, RSA, predictive, etc.) and describe essential details of the model at the first and second levels (e.g. fixed, random or mixed effects; drift or auto-correlation).*
Effect(s) tested	*Define precise effect in terms of the task or stimulus conditions instead of psychological concepts and indicate whether ANOVA or factorial designs were used.*

Specify type of analysis: ☐ Whole brain ☐ ROI-based ☐ Both

Statistic type for inference

(See Eklund et al. 2016)

Specify voxel-wise or cluster-wise and report all relevant parameters for cluster-wise methods.

Correction

Describe the type of correction and how it is obtained for multiple comparisons (e.g. FWE, FDR, permutation or Monte Carlo).

Models & analysis

n/a	Involved in the study
☐	☐ Functional and/or effective connectivity
☐	☐ Graph analysis
☐	☐ Multivariate modeling or predictive analysis

Functional and/or effective connectivity

Report the measures of dependence used and the model details (e.g. Pearson correlation, partial correlation, mutual information).

Graph analysis

Report the dependent variable and connectivity measure, specifying weighted graph or binarized graph, subject- or group-level, and the global and/or node summaries used (e.g. clustering coefficient, efficiency, etc.).

Multivariate modeling and predictive analysis

Specify independent variables, features extraction and dimension reduction, model, training and evaluation metrics.
